# Impact of the COVID-19 Pandemic on the Physical Activity Profile and Glycemic Control Among Qatari Adults With Type 1 Diabetes: Effect of Vaccination Status

**DOI:** 10.3389/fpubh.2022.914117

**Published:** 2022-07-12

**Authors:** Georges Jabbour, Souhail Hermassi, Nicola Bragazzi

**Affiliations:** ^1^Department of Physical Education, College of Education, Qatar University, Doha, Qatar; ^2^Laboratory for Industrial and Applied Mathematics, Department of Mathematics and Statistics, York University, Toronto, ON, Canada; ^3^Department of Health Sciences (DISSAL), Postgraduate School of Public Health, University of Genoa, Genoa, Italy

**Keywords:** COVID-19, type 1 diabetes, physical activity, vigorous physical activity, glycemic control, fear of being infected by COVID-19, vaccine, Qatari adults

## Abstract

**Objective:**

To investigate the impact of COVID-19, as an influent barrier on physical activity (PA) patterns and glycemic control in Qatari adults with type 1 diabetes (T1D). As the COVID-19 vaccine may have a potential impact on an individual's lifestyle, we also considered this parameter.

**Methods:**

Physical activity level, the exercise barriers (BAPAD1), anthropometric characteristics, the method of insulin administration, and the last glycated hemoglobin in % were completed by 102 Qatari adults with T1D. Moreover, all patients were asked whether they had “been vaccinated” or had a “fear of being infected by COVID-19”.

**Results:**

For the unvaccinated group, weight, BMI and HbA1c (%) were significantly higher than those of vaccinated group (*p* < 0.01) and engaged in less moderate-to-vigorous PA (MVPA) (*p* < 0.01) per week and had less time in vigorous PA (VPA) (*p* < 0.01). A significant association between VPA levels and BMI (β = −0.36, *p* = 0.02) and HbA1C (%) (β = −0.22; *p* = 0.03) was reported, and “being vaccinated” was significantly associated with MVPA (β = 0.15; *p* = 0.021) and VPA (β = 0.28; *p* = 0.032). A higher “Fear of being infected by COVID-19” score was negatively correlated with reduced PA profiles (R2 = −0.71 for MVPA; R2 = −0.69 for VPA, *p* < 0.01, respectively).

**Conclusion:**

Practicing VPA during the COVID-19 pandemic confer many health benefits for Qatari individual with T1D. As the “Fear of being affected by COVID-19” appeared as a potential barrier to PA practices this latter e.g. PA, could likely not be achieved without the participants being vaccinated.

## Introduction

Optimal glycemic control and body-weight control are important components of improving overall health in individuals with type 1 diabetes (T1D), and both parameters are improved by physical activity (PA) practices (1, 2). Individuals with T1D are at higher risk of developing serious illness from COVID-19 (3–5). This is due to the disruption of their outdoor activities, including the regular practices of exercise and physical activity (PA), as well as daily care routines, including PA practice, which causes difficulties in glycemic management.

As per the American Diabetes Association's (6) guidelines, PA practices are essential for preventing the deleterious effects of T1D (2, 6), and T1D individuals should engage in at least 150 min/week of moderate- to vigorous-intensity aerobic activity, including vigorous muscle-strengthening and bone-strengthening activities at least 3 days/week. Moreover, the addition of structured exercise may potentiate these benefits (7, 8). Despite the known benefits of PA, the numerous physiological (e.g., hypoglycemia and hyperglycemia episodes) and psychological (e.g., fear of hypoglycemia) risks associated with T1D make it challenging to incorporate PA practices (9). In addition to these conventional barriers, assessed mainly by the BAPAD-1 scale (10, 11), we expect that COVID-19's context may itself constitute an additional potential barrier to PA among adults with T1D and may consequently affect their PA level.

The COVID-19 vaccination campaign, as per the case of the State of Qatar {A total of 256,037 individuals received at least one dose of the mRNA-1273 vaccine, and 181,304 completed the two-dose regimen [between 28 December 2020 and 10 May 2021, (12)]}, has led to most people being more knowledgeable about vaccine effectiveness (e.g., preventing severe illness and death), as reported previously by Chemaitelly et al. (12). One recent study of Schwendinger et al. (13) found a significant association of vaccination status with total PA (*p* = 0.011), vigorous PA (*p* = 0.015), and moderate PA (*p* = 0.001) in German speaking countries. For Ghram et al., (14) the availability of a vaccine might therefore help increase PA in the general population.

In that regard, it may be speculated that vaccinated T1D individuals are less restricted in their PA than those who are not vaccinated. Given the paucity of available data on that matter, we sought to explore the association between COVID-19 vaccination status and PA patterns and PA barriers and among individuals with T1D.

Considering that meeting current physical activity guidelines among individuals with T1D is a major concern, exploring the impact of COVID-19, as an influent barrier to PA practices, on PA levels among Qatari adults with T1D may require additional knowledge to engage in PA safely and increase overall PA. From this perspective, the present study aimed to explore the association between perceived barriers, fear of COVID-19, and level of PA and glycemic control among Qatari adults with type 1 diabetes. As the COVID-19 vaccine may have a potential impact on an individual's lifestyle, we also considered this parameter.

## Methods

### Participants and Study Design

**Figure 1:**
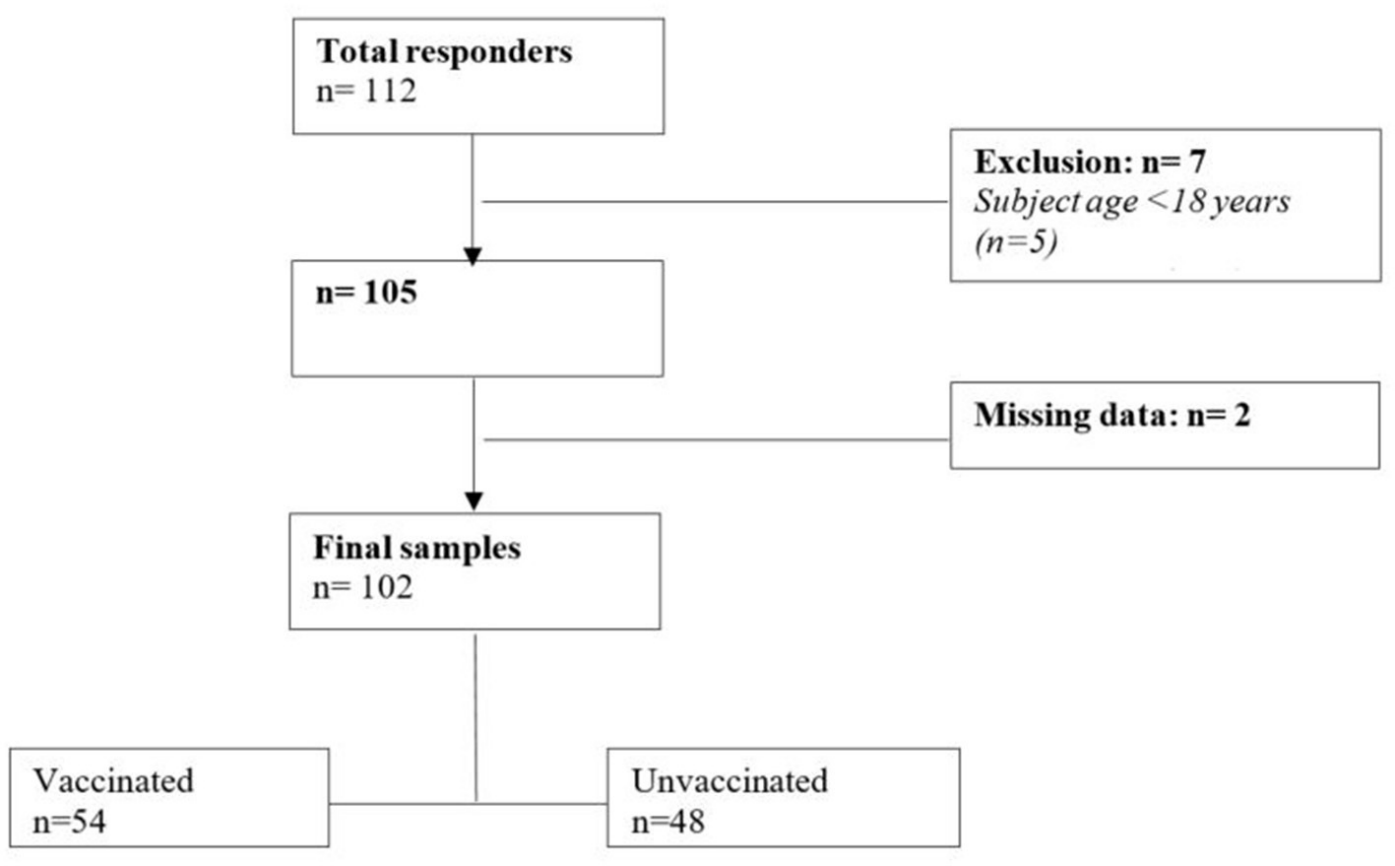
Study design flowchart.

Participants with T1D and of age ≥18 years old were recruited from the State of Qatar. The inclusion criteria were to be Qatari citizen and the capacity to understand English and/or Arabic language. The exclusion criteria were uncontrolled diabetes, comorbidities that do not allow exercise practice, ongoing Covid-19 infection and cognitive impairments. The present study was approved by the Qatar University Institutional Review Board (QU-IRB) (QU-IRB 1559-E/21), and participants were approached and recruited via a link to the electronic survey that has ben distributed via a range of platforms [e.g., Qatar University Announcement to target via email QU community (Students, Faculty Members and Administrators)]. Of the recruited 102 individuals with T1D (Total responders *n* = 112; Figure 1), all agreed to participate in the study and signed an informed consent form according to the QU's code of practice and ethical considerations. All participants completed a round of questionnaires, including (1) the International Physical Activity Questionnaire (IPAQ) and (2) the exercise barriers (BAPAD1) (10, 11). Moreover, anthropometric characteristics such as body weight (kg) and height (m), the method of insulin administration, and the last glycated hemoglobin in % were collected. The method of insulin administration (injection vs. pump) was reported, and patients answered “yes” or “no” to whether they used CGM or engaged in simple self-monitoring with a gluco meter. Moreover, all patients were asked whether they had “been vaccinated” or had a “fear of being infected by COVID-19”. The time period allocated for data collection was two months (October to November 2021). Al the variables of the present study has been analyzed and compared according to vaccination status.

### Activity Profile

To obtain the activity profile, we used the International Physical Activity Questionnaire (IPAQ): The IPAQ assesses the types of intensity of physical activity and sitting time that people engage in during their daily lives and is considered to estimate the total physical activity in min/week and time spent sitting. According to the official IPAQ-SF recommendations, data are summed within each item (i.e., vigorous intensity, moderate intensity, and walking), to estimate the total amount of time engaged in PA per week (15, 16).

### Barriers to Physical Activity

This questionnaire examines 12 barriers to physical activity on a scale from 1 to 7 (1 being extremely unlikely to 7 being extremely likely). This questionnaire examines 12 barriers to physical activity on a scale from 1 to 7 (1 being extremely unlikely to 7 being extremely likely). The BAPAD-1 score was determined by calculating the average of the individual scores obtained for each type of barrier (10, 11).

### Statistical Analysis

The analyses were performed using SPSS v. 21 software (IBM, Armonk, New York, USA). The data are presented as the means (standard deviations). Normality was tested using the Kolmogorov–Smirnov test. The non-normally distributed data were reported as medians and interquartile ranges (IQR). Independent samples t tests were used to assess differences in mean outcome scores within group categories (vaccinated vs. unvaccinated). Pearson product correlation coefficients were determined for relationships between variables that were different among the groups. We used multiple linear regression to model the mean outcomes for each exposure of interest. We used multiple linear regression to model the mean outcomes for each exposure of interest. For both linear and logistic regressions, the independent variables considered in the regression models were total time in moderate to vigorous PA (MVPA) per week in minutes, total time in vigorous PA (VPA) per week in minutes, fear of being infected by COVID-19, and CGM use. Pearson correlations were used to assess the association between “Fear of being infected by COVID-19” and forms of PA, and internal consistency reliability (Cronbach's α-coefficient) for the BAPAD1 scale and “Fear of being infected by COVID-19” was performed; a score of 0.7 and above was deemed acceptable. A value of *p* < 0.05 was set as the level of statistical significance.

## Results

The characteristics of the study participants are presented in Table 1. Among the group, 53% of participants were vaccinated, and 54% of subjects used CGM. However, in the vaccinated group, 95% of participants were using CGM, with 25% in the unvaccinated group. Anthropometric variables (height, weight, BMI percentile) are displayed in Table 1. For the unvaccinated group, weight, BMI and HbA1c (%) were significantly higher than those of the vaccinated group (*p* < 0.01).

**Table 1:** Descriptive characteristics and activity profile of Qatari adults with type 1 diabetes during.

**Study population characteristics**				
	**Total group**	**Vaccinated group**	**Unvaccinated group**	***p*** **-value**
**Percentage of total group (%)**		53%	47%	
Age *(years)* median (Q1–Q3)	24 (18–28)	23.2 (18–29)	22.5 (18–28)	0.52
Duration of diabetes in years, median (Q1–Q3)	14 (11–19)	13 (11–18)	13 (12–20)	0.44
Weight (kg)	74.4 ± 8.6	70.9 ± 14.3^a^	81.8 ± 20.3^ab^	<0.01
Height (cm)	166.2 ± 8.6	167.2 ± 8.9	164.4 ± 9.7	0.58
BMI (kg.m^−2^)	26.9 ± 5.6	25.2 ± 4.9	29.9 ± 5.7^ab^	<0.01
HbA1c (%)	7.6 ± 1.4	6.8 ± 1.1^a^	8.7 ± 1.5^ab^	<0.01
CGM use (%)	54 %	95 %^a^	25 %^ab^	<0.01
Infected by COVID-19 (%)	31 %	31 %	30 %	0.66
**Barriers to Active Lifestyles measured by the BAPAD-1**				
**Diabetes-specific barriers to PA**				
*Loss of control over diabetes*	5.1 ± 1.3	5.1 ± 1.2	4.5 ± 1.5^ab^	<0.01
*Fear of hypoglycemia*	5.16 ± 2.1	5.5 ± 2.1^a^	4.7 ± 2.1^ab^	<0.01
*Fact that you have diabetes*	3.8 ± 1.9	4.1 ± 1.9	3.9 ± 2.3	0.39
*Risk of hyperglycemia*	4.2 ± 1.6	4.3 ± 1.3	3.7 ± 1.8^ab^	<0.01
*Fear of having a heart attack*	2.1 ± 1.6	1.3 ± 0.9	3.4 ± 1.8^ab^	<0.01
**Diabetes-universal barriers to PA**				
*Fear of hurting self*	2.9 ± 1.8	2.8 ± 1.7	2.9 ± 1.6	0.79
*Low fitness level*	4.5 ± 1.8	4.8 ± 1.9	4.3 ± 2.3	0.71
*Weather conditions*	4.1 ±1.3	4.1 ± 1.2	3.9 ± 1.4	0.61
*Sport Center proximity*	3.1 ±1.9	2.9 ± 2.1	3.3 ± 1.8	0.66
*Work schedule*	3.4 ± 2.1	3.1 ± 2.2	4.1 ± 1.6^ab^	<0.01
*Actual physical health status (excluding diabetes)*	4.7 ± 1.9	4.8 ±1.9	4.5 ± 1.6	0.36
Total BAPAD1 score	3.5 ± 1.6	3.5 ± 1.1	3.5 ± 1.4	0.29
Fear of being infected by COVID-19	5.4 ± 1.8	5.9 ± 1.5 ^a^	4.1 ± 1.6^ab^	<0.01
**Self-reported PA and screen time information**				
Total screen time·day^−1^ *(h)* (TV, video games, computer)	6.8 ± 2.66	6.6 ± 2.08	6.3 ± 1.99	0.55
Total time in MVPA. week ^−1^ *(min)*	50.5 ± 10.3	63.6 ± 6.3 ^a^	10.5 ± 3.1^ab^	<0.01
Total time in VPA.week^−1^ *(min)*	30.5 ± 5.3	39.2 ± 3.3 ^a^	3.3 ± 1.1^ab^	<0.01
Total time in Waking.week^−1^ *(min)*	46.1 ± 3.1	58.1 ± 9.9 ^a^	13.3 ± 5.4^ab^	<0.01

*Values are mean (standard deviation). Q1: First quartile; Q3: Third quartile; BMI, body mass index; HbA1c, glycated hemoglobin; CGM, Continuous blood glucose monitoring; PA, physical activity; MVPA, moderate to vigorous physical activity; VPA, vigorous physical activity; h: hour; min: minute*.

*Significant difference with total group (a: P < 0.01)*.

*Significant difference with vaccinated group (b: P < 0.01)*.

The mean BAPAD1 total score was 3.5 ± 1.1 for the vaccinated group and 3.5 ± 1.4 for the unvaccinated group. The internal consistency reliability (Cronbach's α-coefficient) for the BAPAD1 scale and “Fear of being infected by COVID-19” was 0.78 and 0.81, respectively, for vaccinated and unvaccinated adults with T1D. For both vaccinated groups, the highest barrier scores were obtained for loss of control over diabetes and fear of hypoglycemia (Table 1). Moreover, the scores for fear of hypoglycemia and loss of control over diabetes were significantly higher in the vaccinated group than in the unvaccinated group (*p* < 0.01, respectively). Finally, vaccinated individuals reported a higher score for “Fear of being infected by COVID-19” than the unvaccinated group (5.9 ± 1.5 vs. 4.1 ± 1.6, *p* < 0.01). The unvaccinated group engaged in less MVPA (*p* < 0.01) per week and had less time in VPA (*p* < 0.01) and less time in total time in walking per week (*p* < 0.01) than the vaccinated group (Table 1). However, screen activity did not differ statistically among the groups (Table 1).

Our multiple linear regression model showed a significant association between total BAPAD1 score and time spent in all PA forms (Table 2). We also found that “Fear of being infected by COVID-19” was associated with a higher number of barriers (β = 0.44; *p* = 0.033). Moreover, a significant association between VPA levels and BMI (β = −0.36, *p* = 0.02) and HbA1C (%) (β = −0.22; *p* = 0.03) was reported, and “being vaccinated” was significantly associated with MVPA (β = 0.15; *p* = 0.021) and VPA (β = 0.28; *p* = 0.032).

**Table 2:** Standardized regression summary for physical activity time per week, being vaccinated, fear of being infected by COVID-19 and CGM use with other relevant variables.

	**Total MVPA.week^**−1**^ (min)**	**Total VPA.week^**−1**^ (min)**	**Fear of being infected by COVID-19**	**CGM use**
BMI (kg.m^−2^)	R^2^ adj. = 0.041; β = −0.41; *p =* 0.03*	R^2^ adj. = 0.051; β = −0.36; *p =* 0.02*	R^2^ adj. = 0.63; β = 0.05; *p =* 0.77	R^2^ adj. = 0.57; β = 0.04; *p =* 0.48
HbA1c (%)	R^2^adj. = 0.55; β = 0.06; *p =* 0.45	R^2^ adj. = 0.043; β = −0.22; *p =* 0.03*	R^2^ adj. = 0.54;β = 0.04; *p =* 0.22	R^2^ adj. = 0.35;β = 0.07; *p =* 0.53
Total BAPAD1 score	R^2^ adj. = 0.037; β = −0.23; *p =* 0.03*	R^2^ adj. = 0.036; β = −0.14; *p =* 0.05*	R^2^ adj. = 0.051; β = 0.33; *p =* 0.08*	R^2^ adj. = 0.29; β = 0.03; *p =* 0.36
Being Vaccinated	R^2^ adj. = 0.025; β = 0.15; *p =* 0.021*	R^2^ adj. = 0.033; β = 0.28; *p =* 0.032*	R^2^ adj. = 0.047; β = 0.44; *p =* 0.033*	R^2^ adj. = 0.55; β = 0.07; *p =* 0.37

*denotes significant relationship.

A higher “Fear of being infected by COVID-19” score was negatively correlated with reduced PA profiles (R2 = −0.71 for MVPA; R2 = −0.69 for VPA; R2 = −0.88 for walking, *p* < 0.01, respectively) and associated with “being vaccinated” (β = 0.44; *p* = 0.033).

## Discussion

To the best of our knowledge, this study is the first to retrospectively examine the effects of the COVID-19 pandemic on PA levels among T1D adults. While PA practices evaluated subjectively did not meet the current recommendation in terms of PA practices, vaccination status (vaccinated vs. unvaccinated) appeared to affect PA levels in Qatari adults with T1D. Our study indicated that perceived barrier scores were negatively associated with PA levels and that the “Fear of being infected by COVID-19” had the same trend. Moreover, individuals with T1D who were vaccinated were more engaged in VPA and showed better glycemic control, as determined by HbA1c (%), and a better body mass profile than unvaccinated individuals. Because vaccination may confer better protection against COVID-19, vaccinated individuals may have been engaged in more PA than their counterparts. However, vaccinated individuals still accumulated lower PA levels as per the ADA recommendation. Despite the potential role of vaccines in promoting PA practices during the pandemic, other support (psychological management, educational support) is required to overcome the pejorative effect of the pandemic.

This retrospective study of tracked PA during the pandemic showed that Qatari individuals with T1D did not meet the ADA's recommendation in terms of PA practices. A significantly lower level of PA was observed for all forms of PA (MVPA, VPA, and walking activity). In T1D individuals, similar results by Tornese et al. (17) reported a drop in PA that represents a 37% reduction in individuals' weekly minutes of PA. Despite the importance of PA practices, the few data that have explored the effect of pandemics on PA levels in the general population have not established why this pattern was observed (18). However, some evidence from younger populations suggest that being “worried about COVID-19” may be a barrier to PA practices (19), and the increases in sitting time imposed by home confinement may also affect PA levels (20, 21). Our study reported a significant association between PA levels and the “Fear of being infected by COVID-19” and BAPAD1 scores. This result highlights the importance of considering “being infected by COVID-19” as a potential factor in this vulnerable population, especially when addressing strategies to promote PA practices in the COVID-19 context.

Despite the fact that our participants did not meet the current guidelines in terms of PA practices, the interesting results of our study revealed that individuals engaged in more VPA had lower HbA1c levels. Many studies have reported a significant association between exercise intensity and glycemic control, supporting the fact that exercise intensity may lower HbA1c in adults with type 1 diabetes (2, 22). Studies conducted during confinement reported contradictory results. For Di Dalmazi et al. (23) and Pla et al. (24), HbA1c was improved compared with the pre-confinement period, and such a result may be due to more time for self-care and T1D management. However, other studies showed worsening glycemic values that could be due to increased stress and decreased physical activity (25, 26). It is important to note that none of the previous works addressed PA levels or perceived barriers to PA practices or vaccination status as relevant contributing factors. According to our results, the latter was significantly associated with the “being vaccinated” item (β = 0.15; *p* = 0.021 for MVPA; and β = 0.28; *p* = 0.032 for VPA). Despite the fact that we did not investigate the number of vaccination shots, the time they received their vaccine, and other potential confinement-related factors, this study is the first to provide data on a potential role of vaccines in promoting PA practices and therefore lowering glucose levels in Qatari adults with T1D. Having a “Fear of being infected by COVID-19” seems to be a major contributor in vaccine decisions. Getting the vaccine without any reconciliation support to manage this “fear” may be problematic as the COVID-19 situation persists.

The main limitation of our study was that we enrolled a small number of patients with T1D, and therefore, our results may not be extended to all patients with T1D during confinement. Additionally, we did not investigate other potential contributing factors that are known to affect glycemic control, such as nutritional control and behavioral scale scores, and we did not consider any environmental support, which may have a relevance in glucose control. Finally, it must be acknowledged that the data derived from vaccination status did not consider whether T1D participants were on their second or third vaccine doses. Nonetheless, this is the first study providing data on PA levels and glucose control by considering vaccination status and other potential barriers to PA during the COVID-19 pandemic.

In conclusion, although enhancing PA practices during the COVID-19 context may confer important health benefits for T1D individuals, it remains ambiguous “not to say nonexistent” of what PA strategy needs to be implemented during the COVID-19 crisis to promote healthy living. It is necessary to first consider the patients' safety and preoccupation. In that very challenging context, our study highlighted that practicing VPA during the COVID-19 pandemic improved glycemic control as well as body mass index in adults with type 1 diabetes. This outcome could likely not be achieved without the participants being vaccinated. However, it is still challenging to establish the safest strategy to be implemented, especially in regard to considering the current situation that is continually changing as well as the different conditions in adults with T1D.

## Data Availability Statement

The raw data supporting the conclusions of this article will be made available by the authors, without undue reservation.

## Ethics Statement

The studies involving human participants were reviewed and approved by Qatar University Institutional Review Board (QU-IRB) (QU-IRB 1559-E/21). The patients/participants provided their written informed consent to participate in this study.

## Author Contributions

GJ contributed to the conception, design of the study, and drafted the manuscript. GJ, SH, and NB collected data, performed data analysis and interpretation, revised, read and approved the submitted version. All authors contributed to the article and approved the submitted version.

## Funding

This study is supported by a student grant from the Office of Research Support, Qatar University (QUST-1-CED-2022-328).

## Conflict of Interest

The authors declare that the research was conducted in the absence of any commercial or financial relationships that could be construed as a potential conflict of interest.

## Publisher's Note

All claims expressed in this article are solely those of the authors and do not necessarily represent those of their affiliated organizations, or those of the publisher, the editors and the reviewers. Any product that may be evaluated in this article, or claim that may be made by its manufacturer, is not guaranteed or endorsed by the publisher.
